# Full Genome Sequencing of Three *Sedoreoviridae* Viruses Isolated from *Culicoides* spp. (Diptera, Ceratopogonidae) in China

**DOI:** 10.3390/v14050971

**Published:** 2022-05-05

**Authors:** Yingliang Duan, Zhenxing Yang, Glenn Bellis, Jiarui Xie, Le Li

**Affiliations:** 1 Yunnan Tropical and Subtropical Animal Virus Diseases Laboratory, Yunnan Animal Science and Veterinary Institute, Kunming 650224, China; dinlion-yn@163.com (Y.D.); s300yn@163.com (Z.Y.); xjr1990123@163.com (J.X.); 2 Research Institute for the Environment and Livelihoods, Charles Darwin University, Darwin, NT 0909, Australia; glenn.bellis@outlook.com

**Keywords:** *Sedoreoviridae*, BAV, EHDV, *Culicoides*, *C. tainanus*, *C. orientalis*, Yunnan Province, China

## Abstract

*Sedoreoviridae* is a family of viruses belonging to the order *Reovirales* and comprises six genera, two of which, *Orbivirus* and *Seadornavirus*, contain arboviruses that cause disease in humans and livestock. Areas such as Yunnan Province in southwestern China, have high arboviral activity due in part to warm and wet summers, which support high populations of biting flies such as mosquitoes and *Culicoides*. Three viral isolates previously obtained from *Culicoides* collected at cattle farms in Shizong County of Yunnan Province, China, between 2019 and 2020 were completely sequenced and identified as Banna virus (BAV) genotype A of *Seadornavirus* and serotypes 1 and 7 of epizootic hemorrhagic disease virus (EHDV) of *Orbivirus*. These results suggest that *Culicoides* *tainanus* and *C. orientalis* are potential vectors of BAV and EHDV, respectively, and represent the first association of a BAV with *C. tainanus* and of an arbovirus with *C. orientalis*. Analysis using VP9 generally agreed with the current groupings within this genus based on VP12, although the classification for some strains should be corrected. Furthermore, the placement of Kadipiro virus (KDV) and Liao ning virus (LNV) in *Seadornavirus* may need confirmation as phylogenetic analysis placed these viruses as sister to other species in the genus.

## 1. Introduction

Viruses belonging to the *Reovirales* are characterized by possessing multiple genomic segments of dsRNA and a double layer of capsids [1]. Two families, *Sedoreoviridae* and *Spinareoviridae*, are recognized, with *Sedoreoviridae* composed of six genera including two, *Orbivirus* and *Seadornavirus*, which contain arboviruses that cause disease in humans or livestock [2]. The double layers of capsids help these arboviruses to infect diplex hosts, such as mammals and insects [1]. Bluetongue viruses (BTV, the prototype of genus *Orbivirus*), for example, possess an outer capsid protein VP2 and inner capsid protein VP7, which act as ligands binding to the cellular receptors of susceptible ruminant and insect cells, respectively [3,4]. BTV attaches to ruminant host cells via VP2 after they enter the blood through the bite of an infected *Culicoides* vector [3,5]. Following ingestion by the insect vector, virus in the blood meal infects *Culicoides* host cells via VP7 after the outer capsid is digested in the midgut [4,6,7]. Almost all species of *Orbivirus* and *Seadornavirus* are arboviruses [1,5,8,9,10].

Banna virus (BAV), the prototype species of *Seadornavirus* [11], was first isolated from sera and cerebrospinal fluid collected from human patients with encephalitis and fever in Xishuang-Banna of Yunnan Province, China in 1987 [12]. Subsequently, BAV strains have been isolated from cattle and swine [13], and have been reported in Indonesia and Vietnam in addition to China [8,13,14]. Thus far, BAV strains have been isolated from 10 mosquito species belonging to 3 genera (*Aedes*, *Anopheles*, and *Culex*) and unsorted *Culicoides* [13,15,16]. It is uncertain whether BAV can infect ticks, although isolation of BAV from ticks was reported in China in 1992 [17]. Phylogenetic analysis of BAV isolates recognizes four genotypes: A1, A2, B, and C [16,18].

Epizootic hemorrhagic disease virus (EHDV) belongs to the genus *Orbivirus*, and was first recognized from a highly fatal disease of wild white-tailed deer in north America in 1955 [19]. Closely related to BTV, EHDV mainly infects ruminants, especially deer, cattle, sheep and goats [5,20,21,22], and is transmitted by *Culicoides* [23]. EHDV has been reported from Africa, Asia, North America, South America, and Oceania [24]. Thus far, at least seven serotypes (EHDV-1, 2, 4, 5, 6, 7, and 8) are recognized by the World Organization for Animal Health (OIE) [25], while a further two serotypes, EHDV-10 [26,27] and an novel serotype isolated in China [28] reported recently, are yet to be formally recognized. Although EHDV infects bovine, sheep and goats, these hosts are usually asymptomatic or subsymptomatic. Some strains of EHDV-2, EHDV-6 and EHDV-7 have, however, caused several outbreaks of epizootic hemorrhagic disease in deer or cattle in North America and Asia [19,22,29,30,31].

*Culicoides* (Diptera: Ceratopogonidae) is a genus of small biting midges with more than 1340 described species [32]. At least 40 of these are associated with the transmission of approximately 50 animal-associated arboviruses belonging to the families *Peribunyaviridae* (previously known as *Bunyaviridae*), *Sedoreoviridae*, and *Rhabdoviridae* [23,33].

Yunnan Province in the southwest of China encompasses a broad range of climatic zones ranging from tropical areas bordering Myanmar, Laos and Vietnam in the south, to alpine climates bordering Tibet in the north. Warm and rainy summers throughout most of the province are conducive to populations of biting flies such as mosquitoes and *Culicoides*. These factors identify Yunnan as one of the most active provinces in China for insect-borne diseases, particularly if the threat of transboundary diseases from neighboring countries is considered. This high level of arboviral activity is evidenced by the initial discovery in Yunnan of BAV and *Tibet Orbivirus* (TIBOV) [12,34,35], the presence of a large number of strains of BTV and EHDV [28,36,37,38], and the high prevalence of dengue virus in southern Yunnan [39,40,41].

Shizong County of Yunnan Province was the site of the first domestic case of bluetongue disease in China, prompting a number of longitudinal studies into the ecology of this virus in the area [36,42,43]. Duan et al. [35] reported the isolation of three viruses from *C. tainanus* Kieffer and *C. jacobsoni* Macfie in one such study; however, they only identified one of these viruses. This paper reports full genome sequencing of the remaining two viruses and from a third virus isolated from a pool of unsorted *Culicoides* collected in Shizong in 2019 and 2020.

## 2. Materials and Methods

### 2.1. Virus Isolates

Insects were collected from cattle farms at Wulong Village (24.64° N, 104.29° E, 975 m a.s.l.) in Shizong County, then identified, sorted and processed for virus isolation as described previously [35]. Details of two isolates, YNV/01-1 and YNV/03-2, were reported previously [35], while the same methods were used to isolate a third virus from a pool of approximately 100 unidentified parous female *Culicoides* free from visible blood.

### 2.2. Cells

Baby hamster kidney cell line BHK-21 and *Aedes albopictus* cell line C6/36 were cultured in minimum essential medium (MEM) containing 5% fetal bovine serum (FBS), 100 U/mL penicillin and 100 μg/mL streptomycin (Gibco, Thermo Fisher Scientific, Grand Island, NY, USA), under an atmosphere of 95% relative humidity and 5% CO_2_. C6/36 cells were cultured at 28 °C and seeded with viral isolate YNV/01-1, while BHK-21 cells were cultured at 37 °C and seeded with the other two isolates. At viral isolation step performed previously, both cell lines were used to culture potential viruses, respectively.

### 2.3. Extracting Viral Nucleic Acid for Test

An aliquot of 150 μL of supernatant from cell cultures exhibiting obvious cytopathic effect (CPE), was subjected to viral nucleic acid extraction using a MagMAX™-96 viral RNA Isolation kit (Am1836; Ambion, Austin, TX, USA) and a MagMAX™ Express machine (Applied Biosystems, Foster City, CA, USA) following the manufacturers’ directions.

### 2.4. RT-qPCR

RNA samples were denatured at 95 °C and quickly cooled on ice, and then subjected to testing by reverse transcript-quantitative polymerase chain reaction (RT-qPCR). For each sample, 20 μL of reaction solution was prepared using One Step PrimeScript^TM^ RT-PCR kit (Takara, Dalian, China) according to the manufacturer’s instructions, and 4 μL of RNA template, 0.4 μL of each primer and 0.8 μL of probe were added (Table S1). These primers and probes were against Akabane virus (AKAV), BAV, BTV, EHDV, Palyam virus (PALV), and TIBOV, respectively (Table S1). The RT-qPCR was performed on a 7500 Fast Real-time PCR machine (Applied Biosystems, Carlsbad, CA, USA) under the following cycling conditions: 45 °C, 5 min; 95 °C, 10 s; then 95 °C/5 s, 60 °C/34 s for 40 cycles. Fluorescence was measured at the end of each extension step.

### 2.5. Preparing Viral Genomic RNA

Each virus was inoculated to a T75 flask of monolayer cells. YNV/KM3 and YNV/03-2 were inoculated to BHK-21, while YNV/01-1 was inoculated to C6/36. When CPE appeared in 90% of cells at approximately 6 days post-infection, flasks were removed to −80 °C until use.

Cell pellets were collected after scraping cells and centrifugation at 360× *g* for 5 min. Viral genomes were extracted and prepared by methods modified from references [35,44,45]. Briefly, RNA was extracted from cell pellets using RNAiso-plus kit (Takara) according to the manufacturer’s instructions. Air dried RNA precipitant was dissolved by 100 μL of RNase free water, and then an equal volume of 4 M LiCl (Sigma-Aldrich, St. Louis, MO, USA) was added. Single-stranded RNA was removed by precipitation with 2 M LiCl at 4 °C overnight, followed by centrifugation at 15,000× *g* for 2 min. Nearly 200 μL of supernatant was transferred to a new tube and mixed with 500 μL of isopropanol, and then stored at −20 °C for 2 h. The dsRNA was pelleted by centrifugation at 15,000× *g* for 15 min, washed with 1 mL of 75% ethanol, air dried and suspended in 50 μL of RNase free water.

### 2.6. Amplifying Viral Genome

Complete viral genomic cDNA was synthesized by full-length amplification of cDNAs (FLAC) following the methods of Maan et al. [37,46]. Briefly, the 3′ end of the viral dsRNA was covalently linked with an anchor primer by T4 RNA ligase 1 (NEB, USA) at 16 °C overnight. The anchored dsRNA was purified by a MiniBEST Universal RNA Extraction kit (Takara) and used to synthesize cDNA with PrimeScript^TM^ II Reverse Transcriptase kit (Takara) following the manufacturer’s instructions.

Prepared cDNA was denatured at 95 °C and renatured to dsDNA through gradual cooling from 95 °C to 25 °C. Ten microliters of dsDNA and 4 μL of 10 μM primer 5-15-1 [46] were added to a 100 μL PrimeSTAR-GXL (Takara) PCR system and amplified by PCR as described by Duan et al. [37].

### 2.7. DNA Electrophoresis

Viral genomic segments (RT-PCR produced dsDNA) were separated by electrophoresis (90 V for 2–3 h) in 1% agarose gel with dye Goldview II (Solarbio, Beijing, China). Fluorescent bands were screened by a Gel Doc™ XR+ System with Image Lab™ software (Bio-Rad, Hercules, CA, USA).

### 2.8. Complete Sequencing of Viral Genomes

Prepared viral genomic DNA samples were completely sequenced by the MAGIGEN Company (Guangzhou, China) using a HiSeq 2000 system (Illumina, San Diego, CA, USA) and software SOAPdenovo. Sequence data for each segment of each isolate were stored independently on Genbank.

### 2.9. Sequence Data and Phylogenetic Analysis

Public nucleic acid sequences used for analysis in this study were downloaded from GenBank and are listed in Table S2. Complete coding sequence (CDS) regions without stop codons were prepared for phylogenetic analysis. Prepared sequences were aligned by MUSCLE (Codons) with default parameters, and phylogenetic trees were built by Neighbor-Joining (NJ) algorithm (bootstrap = 1000, Model = Kimura 2, d: Transitins + Transversions, Gap Treatment=Pairwise deletion, Codon Position=1) or Maximum Parsimony (MP) algorithm (bootstrap = 1000, Gap treatment = Use all sites, Codon Position = 1) with default parameters. All above operations for analysis were finished using MEGA-11 software. All the phylogenetic trees in this study were constructed by the CDS sequences within viral genomic segments that code corresponding proteins.

## 3. Results

### 3.1. Primary Identification

RNA samples extracted from the supernatants of viral isolates infected cells (YNV/01-1, YNV/KM3 and YNV/03-2), were tested by RT-qPCR using six pairs of primers and probes, respectively (Table S1). As a result, the three viruses were identified as a single BAV and two EHDV (Table 1).

**Figure 1:**
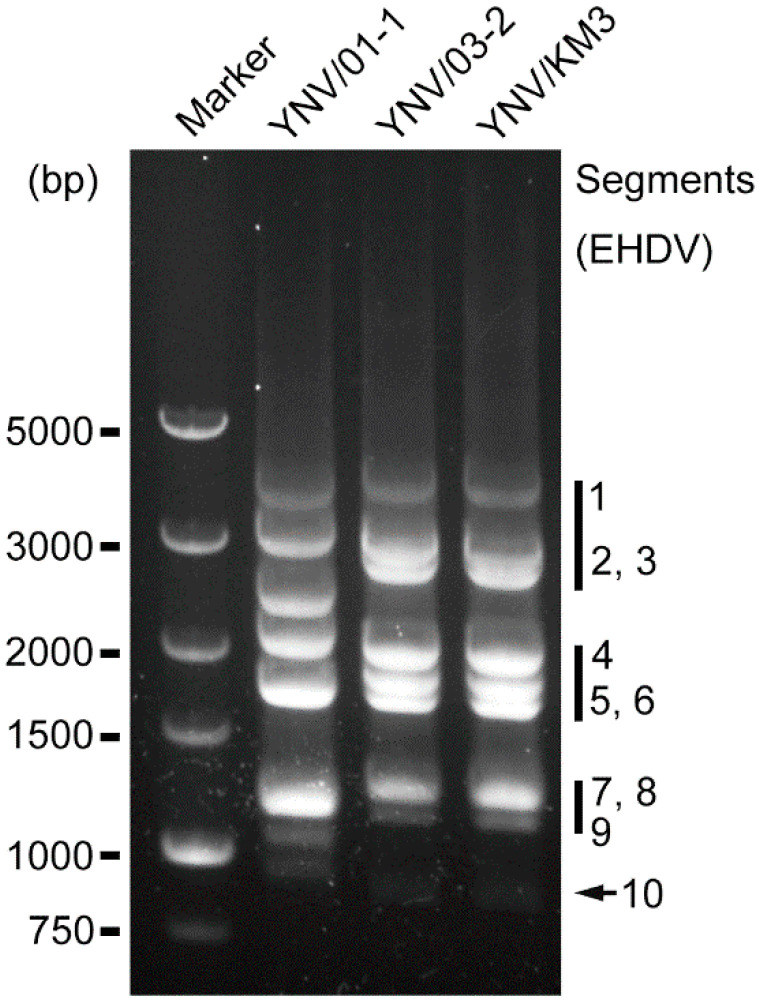
Electrophoresis gel displaying genomic fragments of three viruses isolated from *Culicoides* spp. in Yunnan Province, China. DNA marker and DNA samples of YNV/01-1 (BAV), YNV/03-2 (EHDV-7), and YNV/KM3 (EHDV-1) were separated by electrophoresis in 1% agarose gel. The EHDV segments are annotated on the right.

DNA samples produced by FLAC were subjected to electrophoresis. Segments of BAV (YNV/01-1) were evenly distributed generally, but the 5th band (seg5/seg6), the 6th band (seg7/seg8/seg9), and the 8th band (seg11/seg12) were composed of multiple segments (Figure 1). EHDV isolates YNV/KM3 and YNV/03-2 showed a 3-3-3-1 pattern (Figure 1).

### 3.2. Complete Genome Sequences

Sequence details for each segment of each virus and relevant GenBank accession numbers are provided in Table 2. Sequence data from each virus are provided in Tables S3–S5, respectively. Viral isolates YNV/01-1, YNV/KM3, and YNV/03-2 were identified as BAV, EHDV-1 and EHDV-7 according to their gene sequences (Table 2 and Tables S3–S5). The relationships between viral strains and *Culicoides* hosts are summarized in Table 3.

### 3.3. Phylogenetic Analysis to Confirm Virus Status

To classify the status of genera and viral species, three relatively conservative homofunctional proteins, namely VP1 functioning as a structural protein and RNA dependent RNA polymerase (RdRP), as well as the two major inner capsid proteins on a T = 2 lattice (T2) and a T = 13 lattice (T13), were used for phylogenetic analysis. Difficulties were encountered using NJ and ML algorithms to construct phylogenetic trees of the T13 therefore a MP algorithm was adopted.

**Figure 2:**
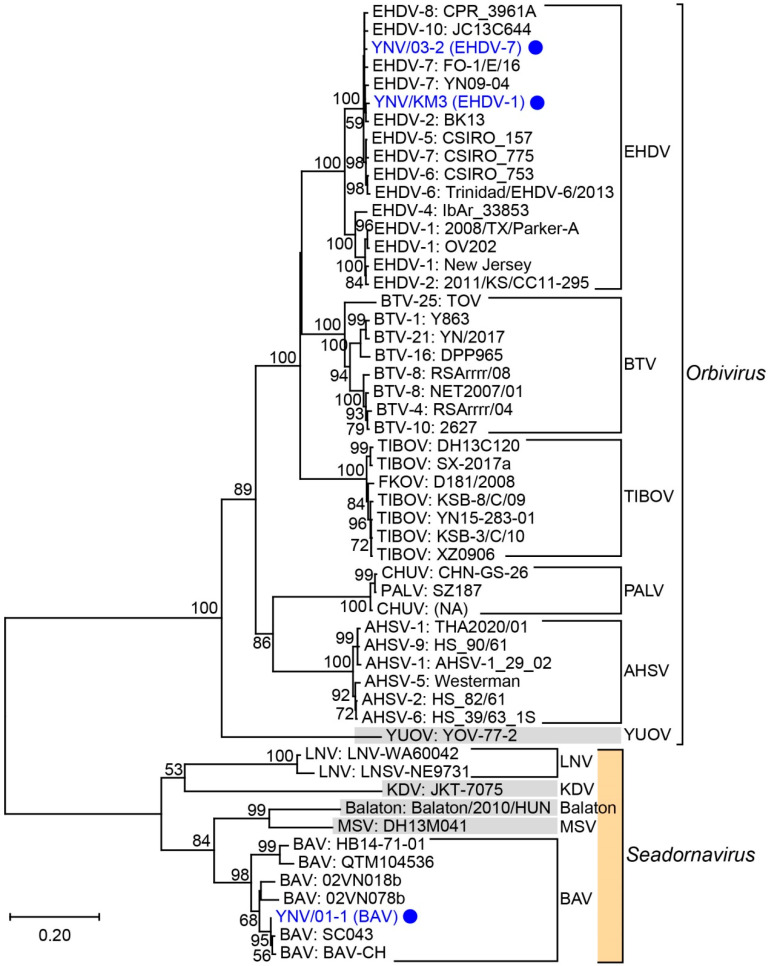
Phylogenetic NJ tree of representative species of *Orbivirus* and *Seadornavirus* based on VP1 (RdRP) genes. Viral species and voucher names are provided; novel isolates reported in this study are highlighted in blue circles. Bootstrap values < 50% omitted.

**Figure 3:**
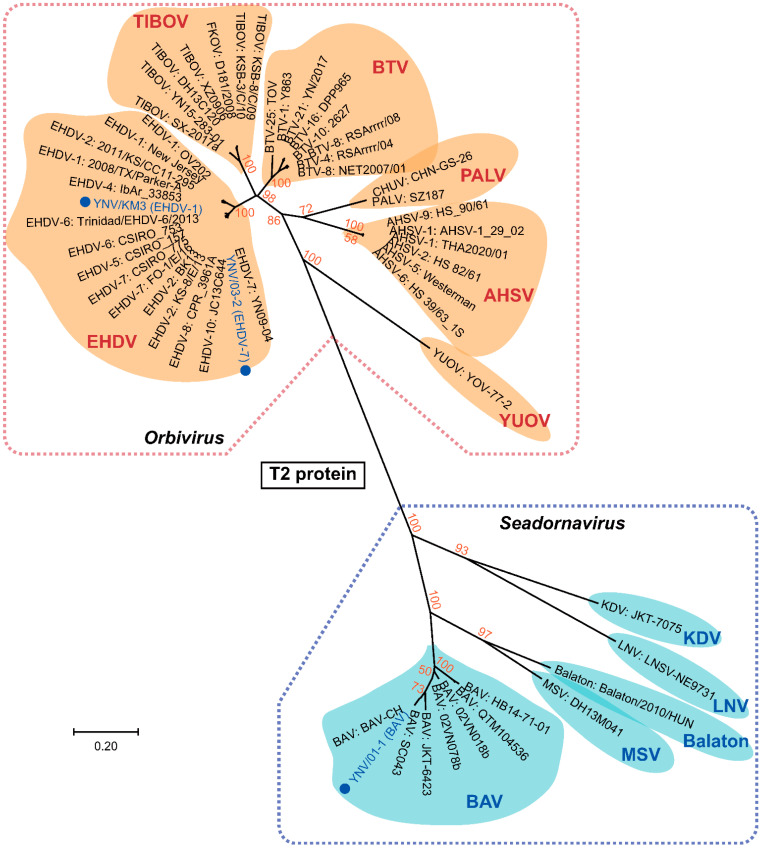
Phylogenetic NJ tree of representative species of *Orbivirus* and *Seadornavirus* based on T2 core capsid protein genes. Viral species and voucher names are provided; novel isolates reported in this study are highlighted in blue circles. Bootstrap values less than 50% are not shown.

In the phylogenetic trees of VP1, T2 proteins, and T13 proteins, *Orbivirus* and *Seadornavirus* and the different virus species were reciprocally monophyletic (Figure 2 and Figure 3 and Figure S1). The status of the BAV (YNV/01-1) and two EHDV (YNV/KM3 and YNV/03-2) isolates reported in this study were confirmed by these phylogenetic trees (Figure 2 and Figure 3, and Figure S1).

The VP1 genetic distance between the nodes of *Orbivirus* and *Seadornavirus* was 0.838, and the shortest distance between an *Orbivirus* (BTV or EHDV) and a *Seadornavirus* (BAV) was 1.316 (Figure 2). The VP1 distances between the clades of *Orbivirus* ranged from 0.192 to 0.692, and the distances between the clades of *Seadornavirus* ranged from 0.301 to 0.896 (Figure 2, Tables S6 and S7).

The T2 protein genetic distance between the nodes of *Orbivirus* and *Seadornavirus* was 0.920, and the shortest distance between an *Orbivirus* (BTV) and a *Seadornavirus* (BAV) was 1.654 (Figure 3). The T2 protein distances between the clades of *Orbivirus* ranged from 0.149 to 0.903, and the distances between the clades of *Seadornavirus* ranged from 0.441 to 1.369 (Figure 3, Tables S8 and S9). Sequences from Kadipiro (KDV) and Liao ning viruses (LNV) were found to be quite distant from other members of *Seadornavirus* such as Balaton virus, BAV, and Mangshi virus (MSV), with genetic distances greater than 1.0 (Table S9). The MP tree of T13 protein (Figure S1) exhibited similar topology to the NJ tree of T2 protein (Figure 3).

### 3.4. Phylogenetic Analysis for Viral Serotypes

**Figure 4:**
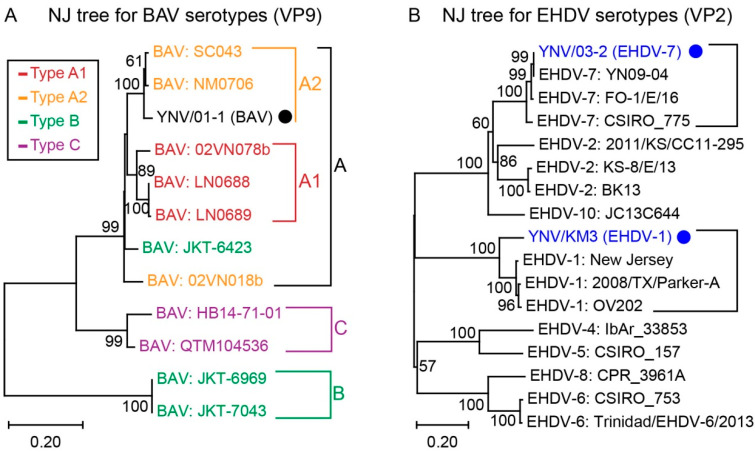
Phylogenetic analysis of BAV genotypes and EHDV serotypes. Phylogenetic trees constructed by NJ algorithm using complete CDS of BAV VP9 (**A**) and EHDV VP2 (**B**), respectively. Viral species and voucher numbers are provided; novel BAV isolate and EHDV isolates reported in this study are labeled by black solid circle and blue solid circles respectively. Bootstrap values < 50% are omitted. In tree (A), previously reported genotypes are highlighted by red (type A1), orange (type A2), green (type B), and violet (type C), respectively.

The phylogenetic tree to classify genotypes of BAV was constructed using VP9 sequences including 11 other BAV belonging to the 4 known genotypes (Figure 4A). In our phylogenetic tree, BAV were grouped into 3 major genotypes (A, B, and C) with VP9 genetic distances between genotypes of 0.719 (A to B), 0.266 (A to C), and 0.727 (B to C), respectively (Figure 4A). There were no clear clades within genotype A, although the so called type A1 and type A2 had a genetic distance of 0.070 (Figure 4A). According to this phylogenetic tree, the novel BAV strain YNV/01-1 reported in this study was grouped with genotype A or A2, however two publicly listed strains, JKT-6423 previously placed within genotype B and 02VN018b previously placed within genotype A2, were in a group that was sister to both the A1 and A2 genotypes (Figure 4A). Extended phylogenetic trees for *Seadornavirus* genotypes, in which VP10 of Balaton virus and LNV as well as VP9 of MSV were added, were constructed by NJ algorithm and MP algorithm, respectively. Both trees supported the above conclusions (Figure S2).

The phylogenetic tree to classify serotypes of EHDV was constructed using VP2 sequences of EHDV (Figure 4B). The two novel EHDV strains, YNV/KM3 and YNV/03-2, were placed in groups with viruses classified as EHDV-1 and EHDV-7, respectively (Figure 4B).

## 4. Discussion

Three viruses isolated from *Culicoides* without blood meals collected from Shizong County of Yunnan Province, China, were identified by full genome sequencing in this study. Viral strain YNV/KM3, isolated from unsorted *Culicoides* species was identified as EHDV, while strains YNV/01-1 and YNV/03-2 isolated from *C. tainanus* and *C. orientalis*, respectively [35], were identified as BAV (genotype A) and EHDV-7, respectively, in this study. The latter two results confirm that BAV and EHDV can infect *C. tainanus* and *C. orientalis*, respectively, and provide the first evidence that these species are potential vectors of these respective viruses.

*Culicoides tainanus* is a widespread species in Yunnan Province, China, and has recently been implicated as a potential important vector of BTV in Asia [47,48,49]. Some evidence has also been reported associating *C. tainanus* with TIBOV, although more evidence is required to confirm this association [35]. The isolation of BAV from this species represents both the third known association with an arbovirus and the first association of BAV with a known species of *Culicoides*.

*Culicoides orientalis* was reported to be a potential vector of *Onchocerca gibsoni* by Buckley [50], but has not previously been associated with the transmission of any virus. In this study, *C. orientalis* was confirmed to be infected by EHDV-7 in the field making this the first report associating *C. orientalis* with EHDV.

Three relatively conservative homofunctional proteins were used for genetic analysis between *Orbivirus* and *Seadornavirus*. VP1 with RdRP function was an essential protein in all viruses analyzed. The other two proteins analyzed, T2 and T13, are the major components of the inner capsid and were both found to provide good resolution to separate viral species within genus *Orbivirus* [51,52]. Primarily, the structure analysis of BTV inner capsid suggested that the icosahedral lattice of inner capsid was constructed by 120 copies of VP3 and 780 copies of VP7, corresponding to a VP3:VP7 composition ratio of 2:13 [53,54,55]. In this study, we tried to construct phylogenetic trees using the homogenous proteins of BTV VP3 (T2) and VP7 (T13) proteins (Figure 3, Figure S1 and Table S2). We, however, encountered problems with constructing both NJ and ML algorithm-based phylogenetic trees for the segments encoding the T13 protein when trying to encompass both the *Orbivirus* and *Seadornavirus* genera. This likely reflected the high variability in structure and function of this protein within each group. T13 proteins of BTV provide the ligand for binding to cellular receptors of susceptible insect cells [4,7] and are consequently likely to evolve to avoid immune responses of insects much the same, as the proteins that provide the ligand for attachment to vertebrate cells are serotype specific, although insect did not produce antibodies [56,57]. Given this selection pressure, genes that code for these proteins in other viruses are also likely to be informative in separating strains of virus, as shown in Figure S1.

Thus far, only three viruses, BAV, KDV, and LNV, have been placed into genus *Seadornavirus* by ICTV [11], and a BAV-like virus (Balaton virus) was reported and considered as a novel *Seadornavirus* by Reuter et al. in 2013 [58]. In the phylogenetic trees of VP1, T2 and T13, KDV and LNV were genetically distant from other species of *Seadornavirus* suggesting that KDV and LNV might be better placed in a novel genus or perhaps, subgenus [8,9,10,58,59].

Existing strains of BAV have traditionally been grouped into four genotypes (A1, A2, B, and C) through phylogenetic analysis of the 12th segment encoding a non-structural protein [13,16,18,60]. However, it is not reliable to classify genotypes or serotypes by non-structural proteins. VP9 of BAV is known as the ligand binding to the cellular receptor of susceptive mammal cells [58,60] so BAV serotypes should be determined by VP9, which is subjected to evolutionary stress from mammalian immune systems such as neutralizing antibodies. This is also more consistent with the method of classifying other arthropod-borne *Reoviruses* like BTV [61]. Our phylogenetic analysis of BAV VP9 found that BAV strains were in general agreement with the system based on VP12, excepting that two viruses were sister to rather than belonging to the A1/A2 clades, and one of these, strain JKT-6423, was placed into genotype A rather than genotype B as previously reported (Figure 4A). However, the VP9/9^th^ segment sequences were lacking for some BAV isolates [13], which compromised the genotype investigation. We encourage future workers to include VP9 data in their descriptions of novel BAV viruses, as this will allow more comprehensive comparison between the classification provided by VP9 and VP12 for serotyping these viruses.

All the known serotypes of EHDV except for EHDV-2 and EHDV-4 have been previously reported in China [28,62,63,64], and seropositive rates of EHDV antibodies are high in cattle in southern China, including Yunnan Province (Duan et al., under review), but no outbreak of clinical disease has yet been reported in Yunnan Province. The only documented outbreak of this disease in mainland China was eight cattle with Ibaraki disease-like symptoms reported in 1987 [65]. In East Asia, a large epidemic of Ibaraki disease in cattle, caused by the Ibaraki strain of EHDV-2, occurred in Japan between 1959 and 1960 [66]; an outbreak of Ibaraki-like disease in cattle in south Japan in 1997 was caused by EHDV-7 strains [26,47].

## Figures and Tables

**Table 1:** Quantification cycle (Cq) values of RT-qPCR tests of three viruses isolated from *Culicoides* spp. in Yunnan Province, China.

Isolate	Cell	Target Virus Species					
		AKAV	BAV	BTV	EHDV	PALV	TIBOV
YNV/01-1	C6/36	NA	16.2	NA	NA	NA	NA
YNV/KM3	BHK-21	NA	NA	NA	26.3	NA	NA
YNV/03-2	BHK-21	NA	NA	NA	27.4	NA	NA

Note: NA means no signal detected, therefore no Cq value.

**Table 2:** Genomic segment lengths and Genbank accession numbers of three viruses isolated from *Culicoides* spp. in Yunnan Province, China.

Seg	YNV/01-1 (BAV)			YNV/KM3 (EHDV-1)			YNV/03-2 (EHDV-7)		
	Length (bp)	Gene	GenBank No.	Length (bp)	Gene	GenBank No.	Length (bp)	Gene	GenBank No.
1	3762	VP1	OM953801	3942	VP1	OM953791	3942	VP1	OM953813
2	3050	VP2 ^(T2)^	OM953802	2968	VP2	OM953792	3002	VP2	OM953814
3	2399	VP3	OM953803	2768	VP3 ^(T2)^	OM953793	2768	VP3 ^(T2)^	OM953815
4	2032	VP4	OM953804	1984	VP4	OM953794	1984	VP4	OM953816
5	1686	VP5	OM953805	1769	NS1	OM953795	1769	NS1	OM953817
6	1671	VP6	OM953806	1640	VP5	OM953796	1641	VP5	OM953818
7	1137	VP7	OM953807	1162	VP7 ^(T13)^	OM953797	1162	VP7 ^(T13)^	OM953819
8	1119	VP8 ^(T13)^	OM953808	1192	NS2	OM953798	1192	NS2	OM953820
9	1100	VP9	OM953809	1071	VP6	OM953799	1074	VP6	OM953821
10	977	VP10	OM953810	810	NS3	OM953800	810	NS3	OM953822
11	867	VP11	OM953811						
12	861	VP12	OM953812						

**Table 3:** Isolation details of three viruses isolated from *Culicoides* collected from Wulong village in Shizong County, Yunnan Province, China.

Isolates	Identification	Hosts	Collection Date	Cells for Isolation	
				C6/36	BHK-21
YNV/KM3	EHDV-1	*Culicoides* spp.	20 August 2019	+	+
YNV/01-1	BAV	*C. tainanus*	20 May2020	+	-
YNV/03-2	EHDV-7	*C. orientalis*	9 June 2020	+	+

## Data Availability

The sequence data are openly available in GenBank (https://www.ncbi.nlm.nih.gov/, accessed on: 22 February 2022).

## Supplementary Materials

The following supporting information can be downloaded at: https://www.mdpi.com/article/10.3390/v14050971/s1, Figure S1: Phylogenetic tree of representative species of *Orbivirus* and *Seadornavirus* based on T13 core capsid protein genes. Figure S2: Phylogenetic trees for genotype analysis of representative *Seadornavirus*. Table S1: Primers and probes used for RT-qPCR tests. Table S2: Data of sequences downloaded from GenBank and used in this study. Table S3: The general genome data of BAV strain YNV/01-1. Table S4: The general genome data of EHDV-1 strain YNV/KM3. Table S5: The general genome data of EHDV-7 strain YNV/03-2. Table S6: VP1 genetic distances between some species of *Orbivirus*. Table S7: VP1 genetic distances between some species of *Seadornavirus*. Table S8: T2 protein genetic distances between some species of *Orbivirus*. Table S9: T2 protein genetic distances between some species of *Seadornavirus*.
