# Supplementary material for: Full Genome Sequencing of Three Sedoreoviridae Viruses Isolated from Culicoides spp. (Diptera, Ceratopogonidae) in China
Source: Viruses. 2022 May 5;14(5):971. doi: 10.3390/v14050971 (PMC9145729; doi:10.3390/v14050971)
Supplement: Supplementary file 1 [file viruses-14-00971-s001.zip › viruses-1679473 supplementary.pdf]

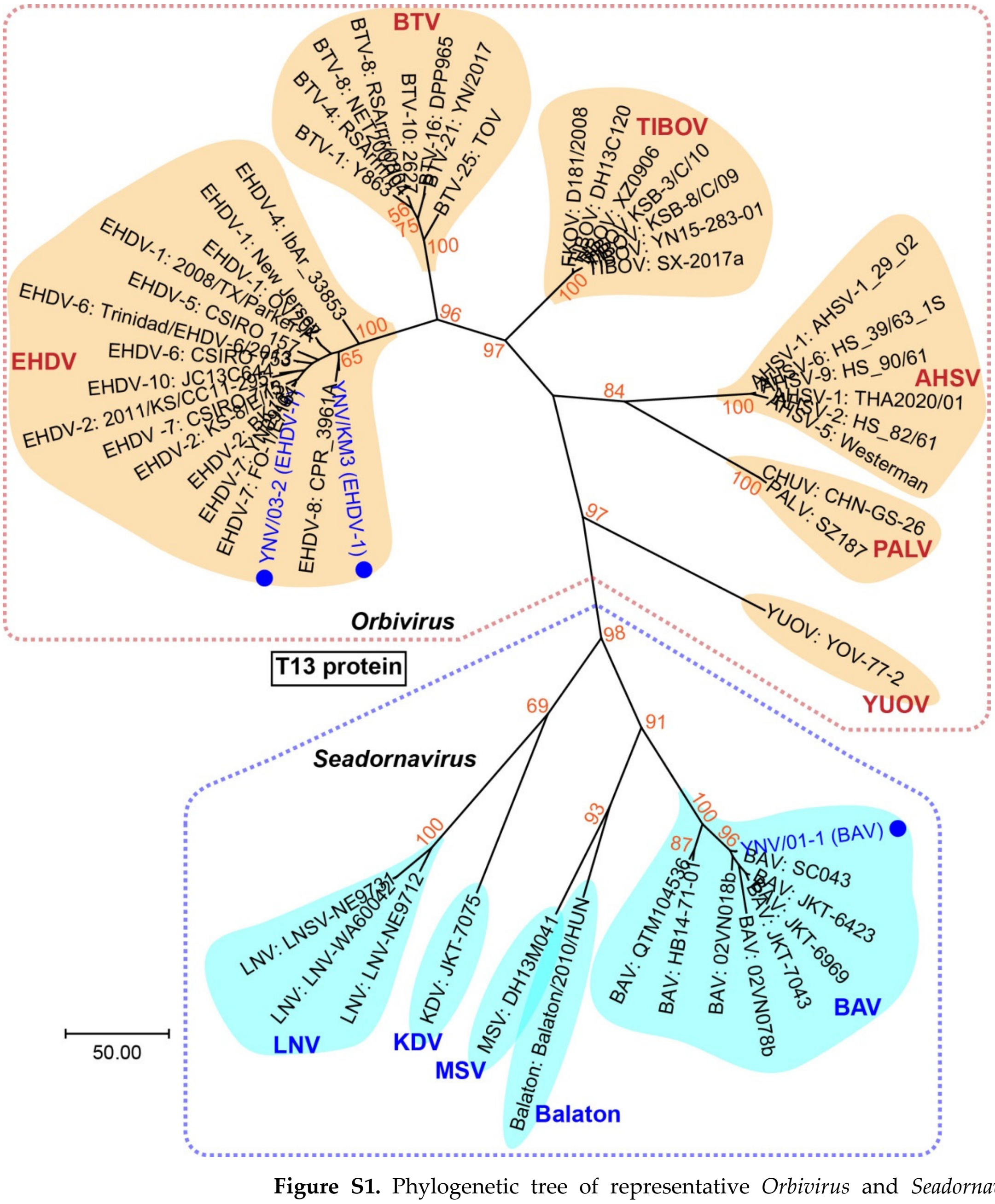

Figure S1. Phylogenetic tree of representative *Orbivirus* and *Seadornavirus* based on T13 core capsid protein genes. This tree is constructed using MP algorithm. Viral species and voucher names are provided; novel isolates reported in this study are highlighted in blue circles. Bootstrap values for major nodes and no less than 50% are shown.

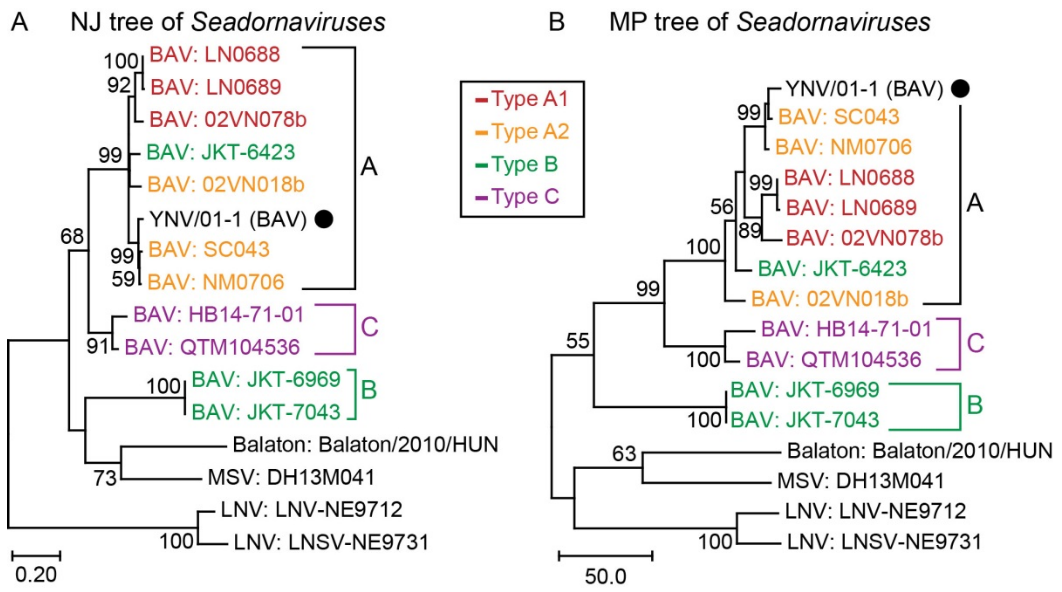

Figure S2. Phylogenetic trees of representative species and genotypes of *Seadornavirus*. Complete CDS of BAV VP9, Balaton virus VP10, MSV VP9, and LNV VP10 are used. Phylogenetic trees are constructed by NJ algorithm (A) and MP algorithm (B) respectively. Previously reported genotypes of BAV are highlighted by red (A1), orange (A2), green (B), and violet (C), respectively.

Table S1. Primers and probes used for RT-qPCR tests

Target virus (gene)	Primer/probe a)	Sequence (5′ to 3′)	Length (bp) b)
AKAV (NP)	AKAV-S-F	AGTATACTGCAAATCCGGTGTCA	102
	AKAV-S-R	TTGATCTGATTAGCCTTGCACTG	
	AKAV-S-probe	CACTGCCTTTACGCTCCAYCGCATCTC	
BAV (VP12)	BAV-S12/YG-F	TTCGCTAGGAGTCAAGGCT	115
	BAV-S12/YG-R	TGCACTGATGCTGGAAATCAAGAA	
	BAV-S12-probe	ACTGTGGGTTGTGAGGGTCCAAGTCA	
BTV (NS3)	BTV-NS3-F	TGGAYAAAGCGATGTCAA A	97
	BTV-NS3-R	ACATCATCACGAAACGCTTC	
	BTV-NS3-probe	ARGCTGCATTCGCATCGTACGC	
EHDV (NS1)	EHDV-NS1-F	TCTTCGTCGACTGCCATCGAG	83
	EHDV-NS1-R	AACATTTTGACATGATTKGCRTARTAACT	
	EHDV-NS1-probe	TGGAGCGCTTTTTGAGAAAATACAACATGA	
PALV (VP7)	PALV-VP7-F	CATCAATGGCAACAATCGGTG	95
	PALV-VP7-R	ATTCAGCATACCTGTAATTCGTAC	
	PALV-VP7-probe	TTCCATATACAACGTCGGCAATGACAAG	
TIBOV (VP6)	TIBOV-S9-F	CTACGGAACGAGGAGGGGAT	100
	TIBOV-S9-R	CTCGCTGCACATTTCCATCTC	
	TIBOV-S9-Probe	ATCAGCTCGTCCTCCTCCCTCTCGT	

a) All the probes are covalently conjugated by fluorescent group FAM (5′) and quenching group BHQ1 (3′). b) Predicted length of segment produced by PCR amplification.

Table S2. Data of sequences downloaded from GenBank and used in this study.

Viruses	Strains	Countries/ regions	Functional genes (segments) and their GenBank access numbers					
			VP1/RdRP	T2 protein	T13 protein	Out ligand	Out capsid	RNA binding
***Orbivirus*:**								
AHSV-1	AHSV-1_29_02	South Africa	S1: KP939370.	S3: KP939380.	S7: KP939400.		S6: KP939395	S8: KP939405.
AHSV-1	THA2020/01	Thailand	S1: MT586213	S3: MT586214.	S7: MT586218		S6: MT586217	S8: MT586219.
AHSV-2	HS_82/61	South Africa	S1: KF859996.	S3: KF859998.	S7: KF860002.		S6: KF860001	S8: KF860004.
AHSV-5	Westerman	South Africa	S1: KP009781.	S3: KP009783.	S7: KP009787.		S6: KP009786	S8: KP009788.
AHSV-6	HS_39/63_1S	South Africa	S1: KF860006.	S3: KF860008.	S7: KF860012.		S6: KF860011	S8: KF860013.
AHSV-9	HS_90/61	South Africa	S1: KF860036.	S3: KF860038.	S7: KF860042.		S6: KF860041	S8: KF860043.
BTV-1	Y863	China	S1: KC879615.	S3: KC879617.	S7: KC879621		S6: KC879620	S8: KC879622.
BTV-4	RSArrrr/04	Cyprus	S1: KP820946.	S3: KP821188.	S7: KP821670.		S6: KP821548	S8: KP821790.
BTV-8	NET2007/01	Netherlands	S1: GQ506451	S3: GQ506453.	S7: GQ506457		S6: GQ506456	S8: GQ506458.
BTV-8	RSArrrr/08	South Africa	S1: KP820956.	S3: KP821198.	S7: KP821680.		S6: KP821558	S8: KP821800.
BTV-10	2627	South Africa	S1: JX272519.	S3: JX272521.	S7: JX272525.		S6: JX272524.	S8: JX272526.
BTV-16	DPP965	Australia	S1: JQ086231.	S3: JQ086233.	S7: JQ086237.		S6: JQ086236.	S8: JQ086238.
BTV-21	YN/2017	China	S1: MK250956	S3: MK250958.	S7: MK250962		S6: MK250961	S8: MK250963
BTV-25	TOV	Switzerland	S1: GQ982522	S3: GQ982523.	S7: EU839843		S6: EU839842	S8: EU839844.
EHDV-1 [a)]	USA2008/TX/Parker-	USA	S1: KU140721	S3: KU140769.	S7: KU140889	S2: KU140745	S6: KU140841	S8: KU140865.
EHDV-1	New Jersey	USA	S1: AM744977	S3: AM744979.	S7: AM744983	S2: AM74497[illegible]	S6: AM744982	S8: AM744984.
EHDV-1	OV202	USA	S1: MF688826	S3: MF688828.	S7: MF688832	S2: MF688827	S6: MF688830	S8: MF688834.
EHDV-2	BK13	Japan	S1: KM509050	S3: KM509052.	S7: KM509056	S2: KM509051	S6: KM509055	S8: KM509057
EHDV-2 [a)]	USA2011/KS/CC11-2[illegible]	USA	S1: KU140706	S3: KU140754.	S7: KU140874	S2: KU140728	S6: KU140824	S8: KU140850.
EHDV-2	KS-8/E/13	Japan	NA	S3: LC202974.	S7: LC599914.	S2: LC202952	S6: LC202963	S8: LC599915.
EHDV-4	IbAr_33853	Nigeria	S1: AM745017	S3: AM745019.	S7: AM745023	S2: AM74501[illegible]	S6: AM745022	S8: AM745024.
EHDV-5	CSIRO_157	Australia	S1: AM745027	S3: AM745029.	S7: AM745033	S2: AM74502[illegible]	S6: AM745032	S8: AM745034.
EHDV-6	CSIRO_753	Australia	S1: AM745037	S3: AM745039.	S7: AM745043	S2: AM74503[illegible]	S6: AM745042	S8: AM745044.
EHDV-6	Trinidad/EHDV-6/201	Trinidad and Tobago	S1: MK919254	S3: MK919256.	S7: MK919260	S2: MK91925[illegible]	S6: MK919259	S8: MK919261
EHDV-7	CSIRO_775	Australia	S1: AM745047	S3: AM745049.	S7: AM745053	S2: AM74504[illegible]	S6: AM745052	S8: AM745054.
EHDV-7	FO-1/E/16	Japan	S1: LC599904.	S3: LC552732.	S7: LC599907.	S2: LC552731	S6: LC552733	S8: LC599908.
EHDV-7	YN09-04	China	S1: MK656453	S3: MK656455.	S7: MK656459	S2: MK65645[illegible]	S6: MK656458	S8: MK656460
EHDV-8	CPR_3961A	Australia	S1: AM745057	S3: AM745059.	S7: AM745063	S2: AM74505[illegible]	S6: AM745062	S8: AM745064.
EHDV-10	JC13C644	China	S1: MT013324	S3: MT013326.	S7: MT013330	S2: MT01332[illegible]	S6: MT013329	S8: MT013331.
PALV	(NA)	Japan	S1: AB018086.	NA	NA	NA	NA	S8: AB018090.
PALV	CHN-GS-26	China	S1: MH090057	S3: MH090060.	S7: MH090069		S6: MF642330	S8: MH090072
PALV	SZ187	China	S1: KT002588.	S3: KT002590.	S7: KT002594		S6: KT002593	S8: KT002595.
TIBOV	D181/2008	China	S1: KR822286.	S3: KR822288.	S7: KR822292		S6: KR822291	S8: KR822293.
TIBOV	DH13C120	China	S1: KU754026	S3: KU754028.	S7: KU754032		S6: KU754031	S8: unusual [b)]
TIBOV	KSB-3/C/10	Japan	S1: LC567102.	S3: LC567104.	S7: LC567108.		S6: LC567107	S8: LC567109.
TIBOV	KSB-8/C/09	Japan	S1: LC567112.	S3: LC567114.	S7: LC567118.		S6: LC567117	S8: LC567119.
TIBOV	SX-2017a	China	S1: NC_033782	S3: NC_033784	S7: NC_03378[illegible]		S6: NC_03378[illegible]	S8: NC_033789
TIBOV	YN15-283-01	China	S1: MT793636	S3: MT793638.	S7: MT793642		S6: MT793641	S8: MT793643.
TIBOV	XZ0906	China	S1: KF746187.	S3: KF746189.	S7: KF746193.		S6: KF746192	S8: KF746194.
YUOV	YOV-77-2	China	S1: AY701509.	S2: AY701510.	S8: AY701516.		S6: AY701514	S7: AY701515.
***Seadornavirus*:**								
Balaton	Balaton/2010/HUN	Hungary	S1: JX947843.	S2: KF790697.	S9: KC522612	S10: JX94784[illegible]	S4: JX947845.	S11: JX947849.
BAV	02VN018b	Vietnam	S1: EU265683.	S2: EU265684.	S8: EU265690	S9: EU265691	S4: EU265686	S12: EU265694
BAV	02VN078b	Vietnam	S1: EU265695.	S2: EU265696.	S8: EU265702	S9: EU312980	S4: EU265698	S12: EU265705
BAV	BAV-CH	China	S1: AF168005.	S2: AF134526.	NA [c)]	NA [c)]	S4: AY549308	NA [c)]
BAV	GS07KD12	China	NA	NA	NA	NA	NA	S12: GQ331954
BAV	GS07KD16	China	NA	NA	NA	NA	NA	S12: GQ331956
BAV	HB14-71-01	China	S1: MH521264	S2: MH521265.	S8: MH521271	S9: MH52127[illegible]	S4: MH521267	S12: MH52127[illegible]
BAV	JKT-6423	Indonesia	S1: unusual [d)]	S2: NC_004217	S8: AF052017.	S9: AF052016	S4: NC_00421[illegible]	S12: AF019908
BAV	JKT-6969	Indonesia	S1: unusual [e)]	S2: unusual [e)]	S8: AF052012.	S9: AF052011	NA	S12: AF052008
BAV	JKT-7043	Indonesia	S1: unusual [e)]	S2: unusual [e)]	S8: AF052028.	S9: AF052027	NA	S12: AF052024
BAV	LN0688	China	NA	NA	NA	S9: KU744434	NA	S12: FJ217990.
BAV	LN0689	China	NA	NA	NA	S9: KU744435	NA	S12: FJ217991.
BAV	NM0706	China	NA	NA	NA	S9: KU744415	NA	S12: GQ331973
BAV	QTM104536	China	S1: KX884638	S2: KX884639.	S8: KX884645	S9: KX884646	S4: KX884641	S12: KX884648
BAV	SC043	China	S1: KC954611.	S2: KC954612.	S8: KC954618	S9: KC954619	S4: KC954614	S12: KC954622
BAV	YN0659	China	NA	NA	NA	NA	NA	S12: FJ161965.
KDV	JKT-7075	Indonesia	S1: NC_00421[illegible]	S2: NC_004212	S9: NC_00420[illegible]		S4: NC_00421[illegible]	S8: NC_004208
LNV	LNV-NE9712	China	NA	NA	S8: AY701346.		S4: AY701342	S11: AY701349
LNV	LNSV-NE9731	China	S1: AY317099.	S2: AY317100.	S8: AY317106.		S4: AY317102	S11: AY317109
LNV	LNV-WA60042	Australia	S1: MG725860	partial [f)]	S8: MG725867		S4: MG725863	S11: MG72587[illegible]
MSV	DH13M041	China	S1: KR349187.	S2: KR349188.	S7: KR349193	S9: KR349195	S4: KR349190	S11: KR349197

a) Full strain names of these viruses are EHD1/USA2008/TX/Parker-A and EHD2/USA2011/KS/CC11-29 respectively. b) This sequence (S8: KU754033.1) of TIBOV strain DH13C120 contains an extra stop codon in CDS, so it was not used. c) Some sequences (S8: AF052034.1; S9: AF052033.1; S12: AF052030.1) were used as the genes of BAV strain BAV-CH in some references, but these records did not shown any strain information in GenBank. d) This sequence (S1: NC_004211.1) of BAV strain JKT-6423 contains many extra stop codons in CDS, so it was not used. e) These sequences from BAV strains JKT-6969 and JKT-7043 were incomplete and strange, so they were not used. f) This sequence (S2: MG725861.1) from LNV strain LNV-WA60042 was incomplete, so it was not used.

Table S3. The general genome data of BAV strain YNV/01-1.

Segments	Encoded proteins and their functions	Segments and genes				Proteins		GenBank accession numbers
		Length (bp)	G+C (%)	CDS ranges	Terminal sequences (5'-3')	Amino acid (aa)	Wt (kDa)	
Seg1	VP1: core capsid, RdRP	3,762	38.44	24: 3683	GUAUUA---ACUGAC	1,219	138	OM953801
Seg2	VP2: core capsid (T2)	3,050	40.39	93: 2960	GUAUAA---CUCGAC	955	108	OM953802
Seg3	VP3: core capsid, guanylyltransferase	2,399	38.02	24: 2186	GUAUUA---ACUGAC	720	82	OM953803
Seg4	VP4: outer capsid	2,032	39.62	27: 1913	GUAUUU---UUUGAC	628	70	OM953804
Seg5	VP5: NS protein	1,686	39.15	32: 1528	GUAUUA---ACUGAC	498	55	OM953805
Seg6	VP6: NS protein, NTPase	1,671	41.35	112: 1389	GUAUUU---ACUGAC	425	48	OM953806
Seg7	VP7: NS protein, protein kinase	1,137	36.41	65: 985	GUAUUA---ACUGAC	306	35	OM953807
Seg8	VP8: core capsid (T13)	1,119	43.25	33: 941	GUAUUU---ACCGAC	302	33	OM953808
Seg9	VP9: outer capsid, cell attachment	1,100	38.91	24: 875	GUAUUU---ACCGAC	283	30	OM953809
Seg10	VP10: core protein	977	37.26	29: 778	GUAUUU---ACUGAC	249	29	OM953810
Seg11	VP11: NS protein	867	39.10	77: 619	GUAUUA---ACUGAC	180	21	OM953811
Seg12	VP12: NS protein, binding dsRNA	861	37.51	44: 667	GUAUUA---ACAGAC	207	24	OM953812
Total		20,661	39.19		GUAU-------------GAC			

Table S4. The general genome data of EHDV-1 strain YNV/KM3.

Segments	Encoded proteins and their functions	Segments and genes				Proteins		GenBank accession numbers
		Length (bp)	G+C (%)	CDS ranges	Terminal sequences (5'-3')	Amino acids (aa)	Wt (kDa)	
Seg1	VP1: core capsid, RdRP	3,942	40.03	12: 3920	GUUAAAAT---ACACUUAC	1,302	150	OM953791
Seg2	VP2: outer capsid, ruminant cell ligand	2,968	40.16	17: 2932	GUUAAAUU---ACACUUAC	971	113	OM953792
Seg3	VP3: core capsid protein (T2)	2,768	41.69	18: 2717	GUUAAAUU---ACACUUAC	899	103	OM953793
Seg4	VP4: core capsid protein	1,984	41.23	9: 1943	GUUAAAAC---CACCUUAC	644	76	OM953794
Seg5	NS1: NS protein, forming tubulin	1,769	43.58	32: 1687	GUUAAAAA---ACACUUAC	551	65	OM953795
Seg6	VP5: outer capsid	1,640	42.87	25:1611	GUUAAAAA---ACACUUAC	528	59	OM953796
Seg7	VP7: core capsid (T13), insect cell ligand	1,162	43.80	18: 1067	GUUAAAAT---CAACUUAC	349	38	OM953797
Seg8	NS2: NS protein, binding dsRNA	1,192	44.71	20: 1147	GUUAAAAA---ACACUUAC	375	44	OM953798
Seg9	VP6: capsid, helicase	1,071	45.28	15: 1025	GUUAAAAA---AAACUUAC	336	37	OM953799
Seg10	NS3: NS protein, virus release	810	43.95	21: 707	GUUAAAAA---ACACUUAC	228	25	OM953800
Total		19,306	41.95		GUUAAA-------------CUUAC			

Table S5. The general genome data of EHDV-7 strain YNV/03-2.

Segments	Encoded proteins and their functions	Segments and genes				Proteins		GenBank accession numbers
		Length (bp)	G+C (%)	CDS ranges	Terminal sequences (5'-3')	ımino acid (aa)	Wt (kDa)	
Seg1	VP1: core capsid, RdRP	3,942	39.73	12: 3920	GUUAAAAU---ACACUUAC	1,302	150	OM953813
Seg2	VP2: outer capsid, ruminant cell ligar	3,002	40.37	18: 2966	GUUAAAUU---AGUCUUAC	982	115	OM953814
Seg3	VP3: core capsid protein (T2)	2,768	42.16	18: 2717	GUUAAAUU---ACACUUAC	899	103	OM953815
Seg4	VP4: core capsid protein	1,984	41.28	9: 1943	GUUAAAAC---CACCUUAC	644	76	OM953816
Seg5	NS1: NS protein, forming tubulin	1,769	42.91	32: 1687	GUUAAAAA---ACACUUAC	551	65	OM953817
Seg6	VP5: outer capsid	1,641	43.69	28:1611	GUUAAAAA---CCACUUAC	527	59	OM953818
Seg7	VP7: core capsid (T13), insect cell ligand	1,162	42.86	18: 1067	GUUAAAAU---CAACUUAC	349	38	OM953819
Seg8	NS2: NS protein, binding dsRNA	1,192	44.97	20: 1147	GUUAAAAA---ACACUUAC	375	44	OM953820
Seg9	VP6: core capsid, helicase	1,074	46.28	15: 1028	GUUAAAAA---AAACUUAC	337	38	OM953821
Seg10	NS3: NS protein, virus release	810	45.06	21: 707	GUUAAAAA---ACACUUAC	228	25	OM953822
Total		19,344	42.06		GUUAAA-------------CUUAC			

Table S6. VP1 genetic distances between species of *Orbivirus.*

	BTV	EHDV	PALV	TIBOV	YUOV
AHSV	0.417	0.417	0.397	0.466	0.653
BTV		0.192	0.456	0.249	0.634
EHDV			0.456	0.249	0.634
PALV				0.505	0.692
TIBOV					0.683

Note: These genetic distances were calculated based on phylogenetic tree of VP1 (Figure 2).

Table S7. VP1 genetic distances between species of *Seadornavirus.*

	BAV	KDV	LNV	MSV
Balaton	0.366	0.896	0.706	0.301
BAV		0.696	0.506	0.349
KDV			0.694	0.879
LNV				0.689

Note: These genetic distances were calculated based on phylogenetic tree of VP1 (Figure 2).

Table S8. T2 protein genetic distances between species of *Orbivirus.*

	BTV	EHDV	PALV	TIBOV	YUOV
AHSV	0.423	0.432	0.393	0.475	0.894
BTV		0.149	0.432	0.192	0.801
EHDV			0.441	0.191	0.810
PALV				0.484	0.903
TIBOV					0.853

Note: These genetic distances were calculated based on phylogenetic tree of T2 proteins (Figure 3).

Table S9. T2 protein genetic distances between species of *Seadornavirus.*

	BAV	KDV	LNV	MSV
Balaton	0.576	1.273	1.369	0.441
BAV		1.029	1.125	0.567
KDV			0.962	1.264
LNV				1.360

Note: These genetic distances were calculated based on phylogenetic tree of T2 proteins (Figure 3).
